# Provision of NICE-recommended varicose vein treatment in the NHS

**DOI:** 10.1093/bjs/znac392

**Published:** 2022-11-30

**Authors:** Louise H Hitchman, Abduraheem Mohamed, George E Smith, Sean Pymer, Ian C Chetter, James Forsyth, Daniel Carradice

**Affiliations:** Academic Vascular Surgery Unit, Hull York Medical School, Hull, UK; Academic Vascular Surgery Unit, Hull York Medical School, Hull, UK; Academic Vascular Surgery Unit, Hull University Teaching Hospitals NHS Trust, Hull, UK; Academic Vascular Surgery Unit, Hull York Medical School, Hull, UK; Academic Vascular Surgery Unit, Hull University Teaching Hospitals NHS Trust, Hull, UK; Academic Vascular Surgery Unit, Hull York Medical School, Hull, UK; Academic Vascular Surgery Unit, Hull University Teaching Hospitals NHS Trust, Hull, UK; Academic Vascular Surgery Unit, Hull York Medical School, Hull, UK; Academic Vascular Surgery Unit, Hull University Teaching Hospitals NHS Trust, Hull, UK; Department of Vascular Surgery, Leeds University Teaching Hospital NHS Trust, Leeds, UK; Academic Vascular Surgery Unit, Hull York Medical School, Hull, UK; Academic Vascular Surgery Unit, Hull University Teaching Hospitals NHS Trust, Hull, UK

## Abstract

**Background:**

Standardization of access to treatment and compliance with clinical guidelines are important to ensure the delivery of high-quality care to people with varicose veins. In the National Health Service (NHS) in England, commissioning of care for people with varicose veins is performed by Clinical Commissioning Groups (CCGs) and clinical guidelines have been developed by the National Institute for Health and Care Excellence (NICE CG168). The Evidence-Based Intervention (EBI) programme was introduced in the NHS with the aim of improving care quality and supporting implementation of NICE CG168. The aim of this study was to assess access to varicose vein treatments in the NHS and the impact of EBI.

**Methods:**

CCG policies for the delivery of varicose vein treatments in the NHS in England were obtained from 2017 (before EBI introduction) and 2019 (after EBI introduction) and categorized by two independent reviewers into levels of compliance with NICE CG168. Hospital Episode Statistics data were compared with the NICE commissioning model predictions. A quality-adjusted life-year was valued at £20 000 (Euro 23 000 15 November 2022).

**Results:**

Despite the introduction of the EBI programme, CCG compliance with NICE CG168 fell from 34.0 per cent (64 of 191) to 29.0 per cent (55 of 191). Some 33.0 per cent of CCG policies (63 of 191) became less compliant and only 7.3 per cent (14 of 191) changed to become fully compliant. Overall, 66.5 per cent of CCGs (127 of 191) provided less than the recommended intervention rate before EBI and this increased to 73.3 per cent (140 of191) after EBI. The overall proportion of patients estimated to require treatment annually who received treatment fell from 44.0 to 37.0 per cent. The associated estimated loss in net health benefit was between £164 and 174 million (Euro 188 million and 199 million 15 November 2022) over 3 years. A compliant policy was associated with a higher intervention rate; however, commissioning policy was associated with only 16.8 per cent of the variation in intervention rate (*R*^2^ = 0.168, *P* < 0.001).

**Conclusion:**

Many local varicose vein commissioning policies in the NHS are not compliant with NICE CG168. More than half of patients who should be offered varicose vein treatment are not receiving it, and there is widespread geographical variation. The EBI programme has not been associated with any improvement in commissioning or access to varicose vein treatment.

## Teaser

Before the pandemic, less than one-third of Clinical Commissioning Groups commissioned in line with National Institute for Health and Care Excellence guidance, leading to only 37.0 per cent of patients receiving the recommended treatment for venous disease annually, with widespread geographical variation. The associated loss in net health benefit was between £164 and 174 million over 3 years. National Health Service England national policy intervention was associated with further deterioration in the quality of care.

## Introduction

Varicose veins are a common condition affecting around 40 per cent of adults in the UK^1,2^. Many patients have complications, including pain, swelling, soft tissue injury, and chronic ulceration. In 2012, the National Institute for Health and Care Excellence (NICE) published its clinical guideline on management of varicose veins (CG168)^3^. This guideline assessed the best available evidence, and concluded that treatment of those with symptoms or complications was highly clinically effective and cost-effective. It was recommended that such patients were assessed and offered interventional treatment by a vascular service without delay. A previous study^4^ published in 2018 found that the commissioning policies of the Clinical Commissioning Groups (CCGs) in England were often non-compliant with CG168, creating geographical variation in access to NICE-recommended treatment.

The document Evidence-Based Interventions: Guidance for CCG*s*^5^ was published in November 2018, and identified 17 areas, including intervention for varicose veins, where evidence-based practice was not widely adopted into CCG policies. It was noted that this resulted in inefficient use of healthcare resources, poor clinical outcomes, and public dissatisfaction in a ‘postcode lottery’ for certain treatments. The programme included guidance to reduce patient harm, improve clinical efficacy, and reduce wasted resources. Evidence-Based Intervention (EBI) supported the unaltered implementation of NICE CG168 for varicose veins.

The aims of this study were to evaluate the success of the EBI programme in supporting the implementation of NICE CG168 and reducing healthcare inequality, and to explore the ability of the National Health Service (NHS) to deliver CG168 and meet the population need for treatment.

## Methods

Each CCG policy was acquired from website resources, direct consultation or via freedom of information requests. In the preceding study, CCG policies were assessed between 17 and 24 April 2017, and this determined the pre-EBI programme policy status^4^. This process was repeated between 26 October and 12 December 2019 to determine post-EBI programme policy status. Two independent reviewers recorded the commissioning criteria and then compared results. Any disagreements were resolved by a third reviewer. Between 2017 and 2019, the number of CCGs reduced due to mergers. For these merged CCGs, the previous policy was taken to be the most compliant of the original individual policies from 2017.

Where CCG policies were non-compliant, the criteria from which they deviated from CG168 (*Table 1*) were noted. CCG policies were split into three groups: red, amber, and green. Green CCG policies were fully compliant with NICE guidance. Amber CCG policies were not compliant, for example not allowing the routine treatment of patients with symptomatic uncomplicated varicose veins (class C2) but allowing treatment of those with soft tissue complications (class C3 and above), or they allowed the treatment of C2 varicose veins with some restriction or limitation^6^. Red CCG policies only permitted routine treatment for patients with venous leg ulcer disease (C5 and C6) or acute complications such as bleeding.

**Table 1:** Guidance on patient groups for referral to a vascular service for assessment and interventional treatment for varicose veins in both National Institute for Health and Care Excellence Clinical Guidance 168 and Evidence-Based Intervention

	Clinical feature of varicose veins	NICE recommendation
CEAP C0	No visible/palpable venous disease	Not recommended
CEAP C1	Telangiectasias or reticular veins	Not recommended
CEAP C2	Varicose veins plus symptoms (pain, aching, discomfort, heaviness, itching)	Recommended*
CEAP C3	Lower limb oedema due to CVI	Recommended*
CEAP C4	Pigmentation and/or eczema due to CVI	Recommended*
CEAP C5	Healed varicose leg ulcer	Recommended*
CEAP C6	Varicose ulcer (break in skin below the knee present for ≥2 weeks)	Recommended*
Complications	Bleeding varicose vein	Recommended—immediate*

Do *not* offer compression as a treatment unless patient is unsuitable for interventional treatment. NICE, National Institute for Health and Care Excellence; CEAP, Clinical Etiologic Anatomic Pathophysiologic; CVI, chronic venous insufficiency.

To investigate the impact of EBI, and the association between CCG policy and actual practice, Hospital Episode Statistics (HES) for each CCG in England were acquired from NHS Digital. These data included treatment spells associated with a varicose vein intervention from April 2017 to March 2020 for each CCG. Where the number delivered by a CCG was between 0 and 5, this was documented as 5*, to preclude the possibility of individual-patient identification from the data. For analysis, 5* was taken as 5. The caseload per CCG was standardized as number of interventions per 100 000 patients per year to allow direct population-level comparison.

The NICE costing template was produced as a workstream of NICE CG168^3^. The costing model was developed to aid CCGs with predictions for the number of expected interventions and the associated costs, under the new guidance. This model was based on the available epidemiological data and the local population for each CCG. The template was used to estimate the expected number of varicose vein procedures per CCG by selecting the percentage of the population who were adult and over 18 years old in each CCG.

In determining the deficit/excess intervention rate per CCG, a 10 per cent allowance between the actual and NICE-predicted number of interventions was used. For example, CCGs with intervention rates of between 90 and 110 per cent of their NICE prediction were considered to be providing treatment as expected. CCGs with intervention rates of less than 90 per cent of predicted were considered to be providing less than the expected intervention rate, and those with intervention rates of greater than 110 per cent of predicted were considered to be providing more than the expected rate.

Intervention rates are reported as counts and medians with 95% confidence intervals. Data related to policy are presented as counts and percentages. To explore the relationship between policy, change in policy, and intervention rate, data were checked for normality and entered into a linear regression model.

The overall difference between actual and predicted interventions across England was calculated and the difference in quality-adjusted life-years (QALYs) was estimated using QALY gain data for intervention *versus* conservative management from the National Institute for Health and Care Research REACTIV trial (group 3) for each year (2017–2018, 2018–2019, 2019–2020) as well as over the entire 3-year study interval (April 2017 to April 2020)^7^. The estimation of net health benefit is based on the following assumptions: all procedures were performed at the highest endovascular tariff cost; there were no additional costs of conservative management; patients who were treated conservatively did not have disease progression; and all patients denied varicose vein treatment had symptomatic varicose veins, rather than complications such as ulceration. The perspective was from a third-party healthcare payer and did not consider societal costs. Discounting was not applied to this estimate, and the willingness-to-pay threshold was £20 000 per QALY (Euro 23 000 15 November 2022). The assumed cost of providing a varicose vein intervention was £780 (Euro 892 15 November 2022), based on the NICE costing template of a single consultant operator performing an endovenous ablation. These assumptions were designed to minimize the impact of the treatment deficit and provide the most conservative assessment of the restriction in healthcare provision.

The data were entered into a bespoke spreadsheet and analysed using Microsoft^®^ Excel (Microsoft, Redmond, WA, USA) and SPSS^®^ version 28 (IBM, Armonk, NY, USA). Maps were created in Tableau (Tableau Software, Seattle, WA, USA) using CCG boundaries (April 2019) available at http://geoportal.statistics.gov.uk^8^. Sankey diagrams were generated using Sankeymatic.com^9^.

Ethical approval was not sought as all data are publicly available and anonymized.

### Patient and public involvement

This study aimed to help address the number 1 research priority identified during the James Lind Alliance research Priority Setting Partnership by patients with venous diseases: ‘How can all patients be given the opportunity to access the specialist assessment and treatment they need?’^10^.

## Results

Some 191 CCGs were included in the analysis; 188 had a policy for the treatment of varicose veins in 2017. All CCGs had a policy for the treatment of varicose veins by 2019.

**Fig. 1:**
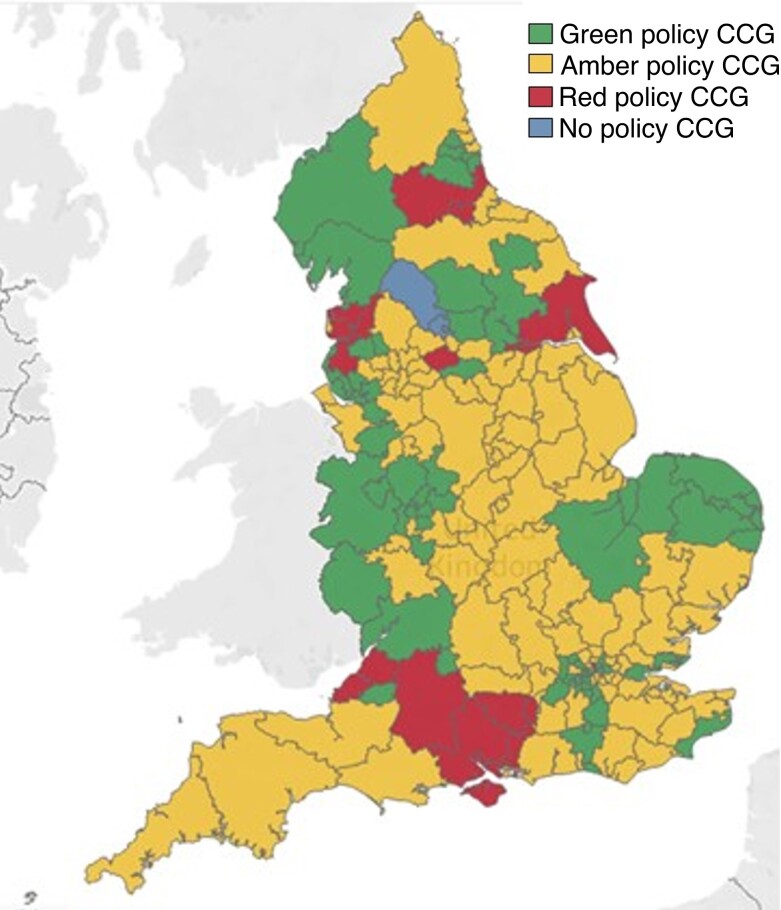
Clinical Commissioning Group compliance with National Institute for Health and Care guidance before Evidence-Based Intervention programme Green, fully compliant; amber, non-compliant but allows treatment in some circumstances; red, treatment only for ulceration or bleeding. CCG, Clinical Commissioning Group.

**Fig. 2:**
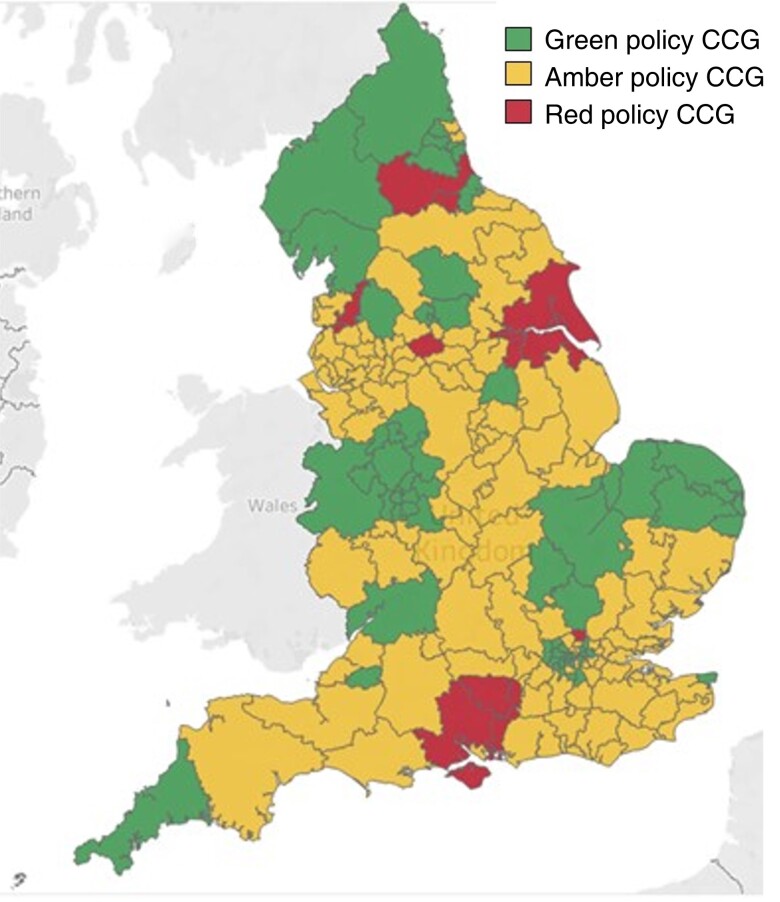
Clinical Commissioning Group compliance with National Institute for Health and Care Excellence guidance after Evidence-Based Intervention programme Green, fully compliant; amber, non-compliant but allows treatment in some circumstances; red, treatment only for ulceration or bleeding. CCG, Clinical Commisioning Group.

**Fig. 3:**
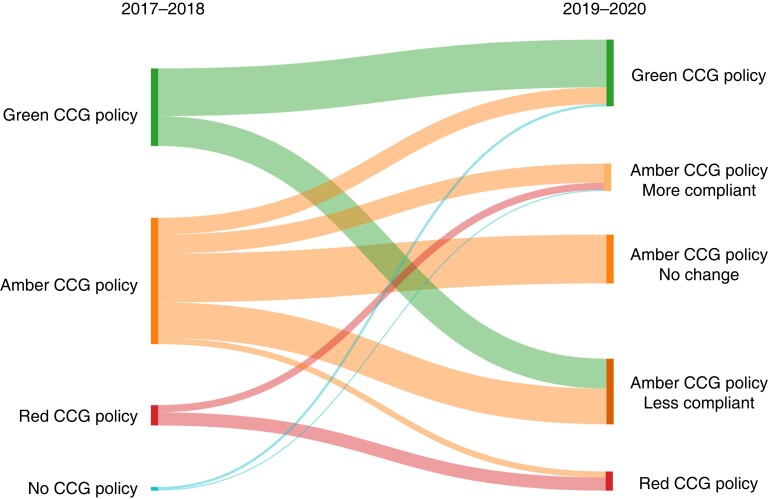
Change in Clinical Commissioning Group compliance with guidance from before (2017–2018) to after (2019–2020) Evidence-Based Intervention programme CCG, Clinical Commissioning Group.

The overall CCG policy compliance rates decreased following EBI from 34.0 to 29.0 per cent (*Figs 1* and *2*). Over this time frame, 21.5 per cent of CCG policies (41 of 191) remained fully compliant, 7.3 per cent (14 of 191) changed to become compliant, 12.6 per cent (24 of 191) became more compliant but fell short of full compliance, 25.7 per cent (49 of 191) remained the same and not fully compliant, and 33.0 per cent (63 of 191) became less compliant than previously (*Fig. 3* and *supplementary material*).

**Fig. 4:**
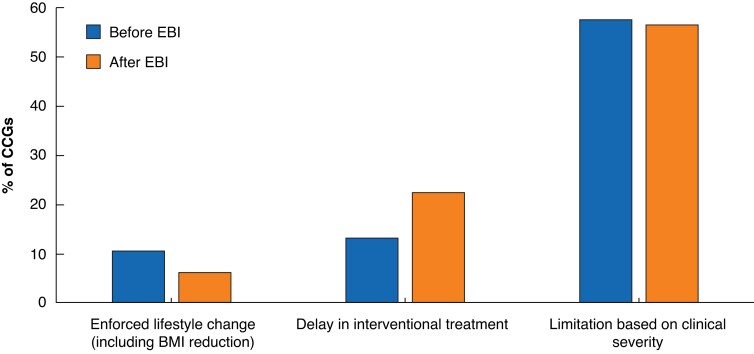
Ways in which Clinical Commissioning Group policies deviated from National Institute for Health and Care Excellence Cinical Guidance 168 CCG, Clinical Commissioning Group; EBI, Evidence-Based Intervention.

**Fig. 5:**
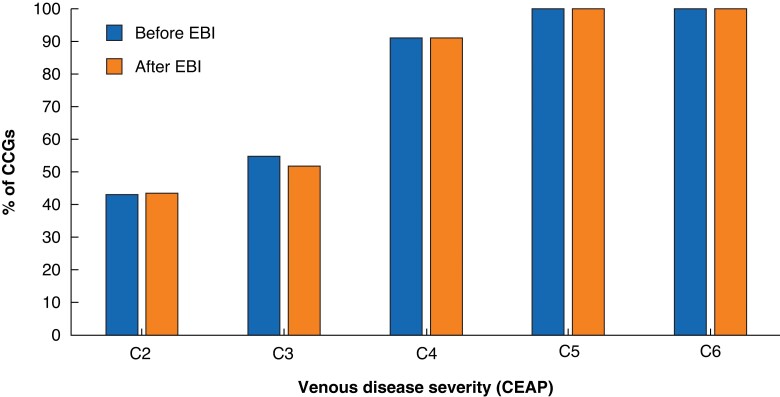
CEAP clinical score of venous disease treated by Clinical Commissioning Groups CCG, Clinical Commissioning Group; CEAP, Clinical Etiologic Anatomic Pathophysiologic; EBI, Evidence-Based Intervention.

The most common deviations from CG168 were limiting treatment to more clinically advanced disease (C4 and above) and delaying intervention for a trial of conservative therapy (*Figs 4* and *5*). In most CCGs, this involved 6 months of compression hosiery before referral to a specialist service. No CCGs prevented the treatment of superficial venous thrombosis.

**Fig. 6:**
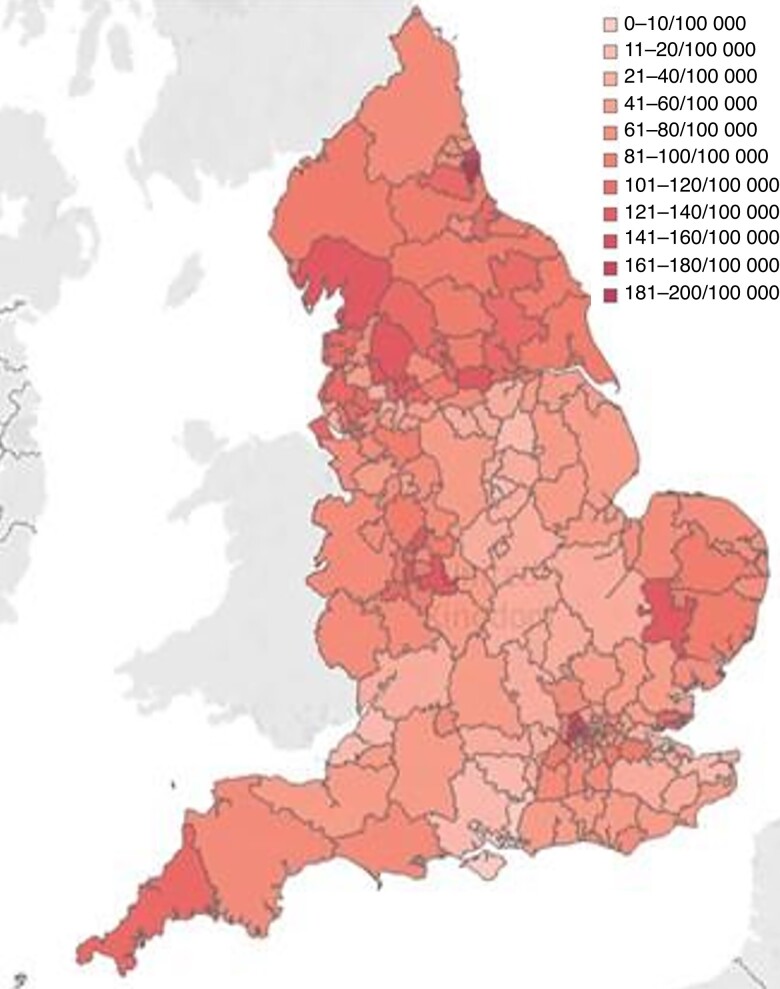
Varicose vein intervention rate by Clinical Commisioning Group in 2017–2018

**Fig. 7:**
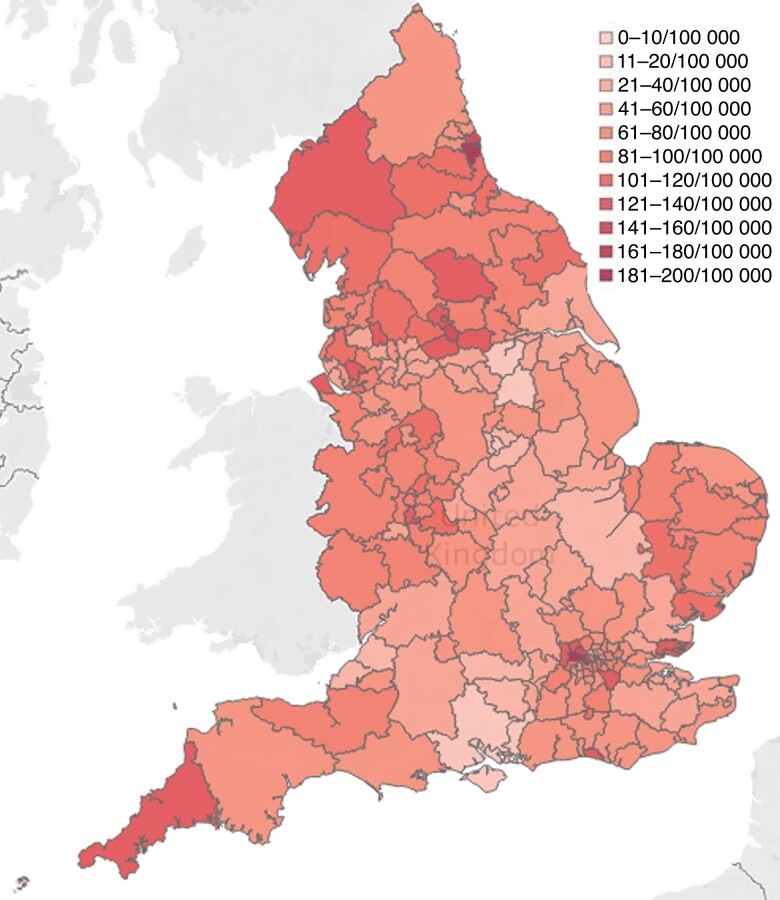
Varicose vein intervention rate by Clinical Commisioning Group in 2019–2020

In 2017–2018, the total number of varicose vein interventions was 30 020. This decreased to 25 770 in 2019–2020 (14.2 per cent reduction). The overall intervention rate in England during 2017–2018 was 68 per 100 000, falling to 59 per 100 000 in 2019–2020. This compares with a NICE-predicted national intervention rate for England of 112 per 100 000. The median intervention rate across CCGs in England declined from 66 (range 4–195) per 100 000 in 2017–2018 to 53 (2–173) per 100 000 in 2019–2020 (*Fig. 6* and *7*). Assuming that all procedures completed were in line with NICE guidelines, there was a deficit of 38 754 procedures in 2017–2018 and 43 040 in 2019–2020, meaning that the proportion of predicted procedures actually performed was 44 and 37 per cent respectively. If all procedures over 110 per cent of predicted for individual CCGs were assumed to be outside of NICE-recommended treatment, the deficit could be as high as 58 and 64 per cent. Overall, before EBI, 9.4 per cent of CCGs (18 of 191) met the predicted intervention rate, 66.5 per cent (127 of 191) did not meet the predicted rate, and 24.1 per cent (46 of 191) exceeded it. After EBI, 11.0 per cent of CCGs (21 of 191) met the predicted intervention rate, 73.3 per cent (140 of 191) did not meet the predicted rate, and 15.7 per cent (30/191) exceeded it.

**Fig. 8:**
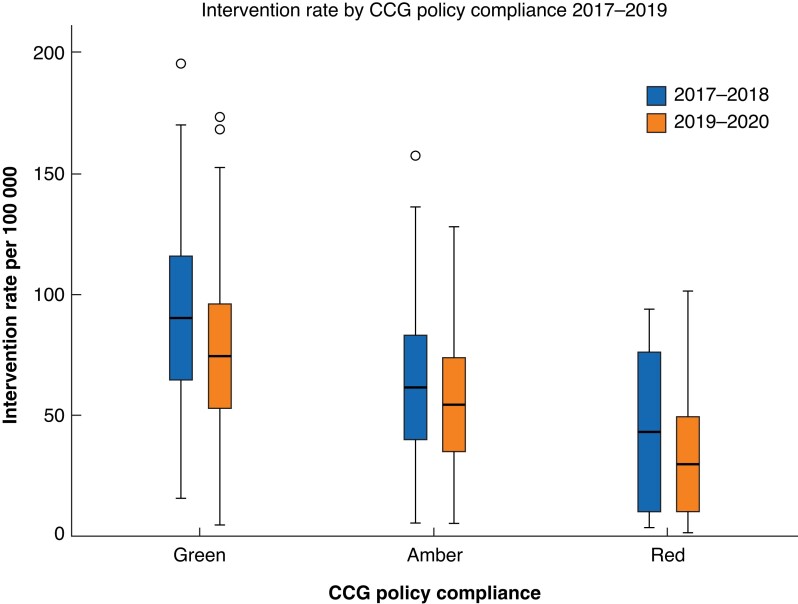
Intervention rate by Clinical Commisioning Group policy compliance group in 2017–2018 and 2019–2020 Median (bold line), (box), and range (error bars) excluding outliers (symbols) are shown. CCG, Clinical Commissioning Group.

**Fig. 9:**
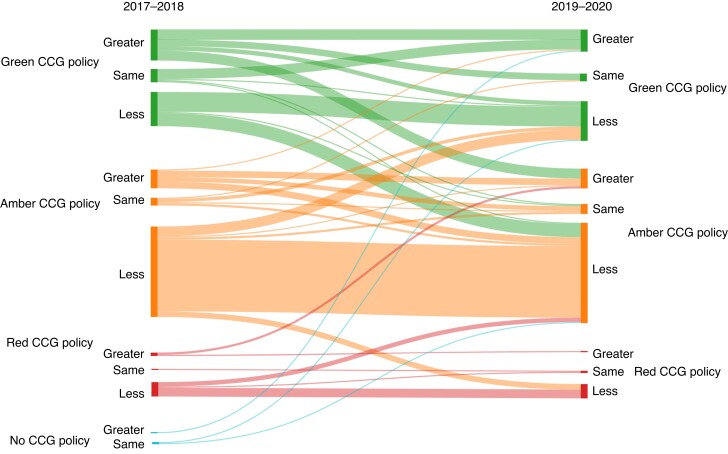
Change in **Clinical Commissioning Group policy compliance and median intervention rate between 2017–2018 and 2019–2020** The green, amber, red, and no policy categories correspond to the Clinical Commissioning Group (CCG) policy compliance; greater, same, and less categories represent the actual *versus* predicted intervention rate in that CCG.

For CCGs with compliant (green) policies, the median intervention rate fell slightly from 78 (range 24–195) per 100 000 in 2017–2018 to 76 (5–173) per 100 000 in 2019–2020 (*Fig. 8* and *supplementary material*). Before EBI, 12 per cent of green CCGs (8 of 65) met the predicted intervention rate, 49 per cent (32 of 65) did not meet the expected rate, and 39 per cent (25 of 65) exceeded it. After EBI, these proportions were 16 per cent (9 of 56), 64 per cent (36 of 56), and 20 per cent (11 of 56) respectively (*Fig. 9* and *supplementary material*).

The fall in intervention rate between 2017–2018 and 2019–2020 was most marked in the amber and red CCG policy compliancy groups (*Fig. 8*). In the amber group, the median intervention rate fell from 56 (range 6–168) per 100 000 to 48 (6–128) per 100 000 (*Fig. 7*). Before EBI, 7 per cent of amber CCGs (7 of 106) met the predicted intervention rate, 77 per cent (82 of 106) did not, and 16 per cent (17 of 106) exceeded it. After EBI, these proportions were 8 per cent (10 of 119), 75 per cent (89 of 119) and 17 per cent (20 of 119) respectively.

In the red CCG policy compliancy group, the median intervention rate fell from 46 (range 4–109) per 100 000 to 30 (range 2–101) per 100 000 (*Fig. 8*). Before EBI, 6 per cent of red CCGs (1 of 17) met the predicted intervention rate, 76 per cent (13 of 17) did not, and 18 per cent (3 of 17) exceeded it. After EBI, these proportions were 13 per cent (2 of 16), 81 per cent (13 of 16), and 6 per cent (1 of 16) respectively.

Of the three CCGs that did not have a varicose vein treatment policy in 2017, one had a higher than predicted intervention rate, and two were within the predicted range in 2019–2020.

Comparing CCG policy with intervention rate, a compliant policy was associated with a higher intervention rate. Compared with green CCGs, amber CCGs performed 24 (95 per cent c.i. 14 to 33) per 100 000 fewer procedures (*P* < 0.001) and red CCGs performed 44 (27 to 60) per 100 000 fewer (*P* < 0.001). CCG policy status accounted for approximately 17 per cent of the variation in intervention rate (*R*^2^ = 0.168, *P* <0.001).

For CCGs whose policy improved to being fully compliant by 2019–2020, the intervention rate was comparable to that of CCGs with established fully compliant policies (*P* = 0.660). For CCGs whose policies improved but were still not fully compliant, the intervention rate was 20 (95 per cent c.i. 5 to 35) per 100 000 less than that for fully compliant CCGs (*P* = 0.010). For CCGs whose policy did not change and remained non-compliant, the intervention rate was 30 (18 to 43) per 100 000 less than that of fully compliant CCGs (*P* < 0.001). In CCGs with decreasing compliance, the intervention rate was 25 (14 to 37) per 100 000 less than that of fully compliant CCGs (*P* < 0.001). A change in policy accounted for approximately 16 per cent of the change in CCG intervention rate (*R*^2^ = 0.164, *P* < 0.001).

Assuming that all procedures performed were within NICE guidelines, the estimated lost opportunity in annual QALY gain was 2095 in 2017–2018, 2162 in 2018–2019, and 2324 in 2019–2020. Over the whole study, the cumulative estimate of lost QALY gain was 12 931 (March 2017 to March 2020). At a willingness-to-pay threshold of £20 000 per QALY (Euro 23 000 15 November 2022), the value of this deficit was £258 625 559 (Euro 295 836 178 15 November 2022) . The cost of providing the additional procedures would have been an estimated £95 050 106 (Euro 108 725 758 15 November 2022), assuming that all were treated with endothermal ablation. The net loss from this failure to provide treatment in line with NICE guidance is therefore an estimated £163 575 453 (Euro 187 110 419 15 November 2022). This figure will rise exponentially until the issue is addressed.

Assuming that procedures over and above 110 per cent predicted within a CCG were outside of NICE guidance, the cumulative estimate of lost QALY gain increased to 13 456 (March 2017 to March 2020), with an estimated value of £269 118 839 (Euro 307 839 214 15 November 2022). The cost of the additional procedures (outside of NICE guidance £3 533 400 Euro 4 041 780 15 November 2022) would be added to this. The cost of providing the procedures (within NICE guidance) would be £98 583 506 (Euro 112 767 538 15 November 2022). The net estimated loss in this scenario would therefore rise to £174 068 733 (Euro 199 113 455 15 November 2022).

## Discussion

NICE CG168^3^ provided a detailed review of the evidence base, and found varicose vein procedures to be highly clinically effective and cost­-effective for patients with symptoms or complications from superficial venous reflux, and recommended that interventional treatment be offered.

The objective of the NHS EBI programme was to address any discrepancy between evidence-based guidelines, CCG policy, and CCG intervention rates. A stated aim of the EBI programme is ‘to reduce the number of inappropriate interventions carried out by clinicians…and to improve the quality-of-care patients receive’^5^. Regarding the management of patients with varicose veins, there was a clear and unambiguous statement that CCGs should follow NICE CG168^3^. The EBI’s mission is ‘reducing harm to patients, minimising unwarranted variation…and optimising the use of finite NHS resources’^11^. It is important to note that, before the EBI programme, CCGs in England performed 30 020 interventions for varicose veins; this represents only 44 per cent of the NICE-estimated annual procedural numbers with full implementation of the guideline. In this scenario, therefore, the aim of the EBI programme should have been to increase the number of appropriate interventions, improving compliance with NICE CG168.

Unfortunately, this study suggests that the EBI programme was not associated with increasing compliance. Overall CCG policy compliance with NICE CG168 decreased, with the proportion of fully compliant CCGs falling from 34 to 29 per cent. This, however, is not the full story, as 33 per cent of CCGs became more restrictive and less compliant than they were before the EBI programme. This decrease in the quality of commissioning was associated with a 14 per cent fall in procedure numbers, with a treatment deficit of over 43 000 cases. This resulted in an estimated cumulative loss of 12 900–13 500 QALYs over this short study interval, with a net loss in health benefit of £164–174 million. This does not include the full additional healthcare costs, such as community dressings, ongoing use of compression garments and recurrent primary care, and in some instances emergency department attendances. Nor does it include the societal loses in a working age population, with disruption of both employment productivity and caring roles. These costs will escalate exponentially as the lost QALYs will persist and in some instances increase for the lifetime of the population along with the addition of each year’s incident unmet need.

Before the EBI programme, there was significant geographical variation in access to treatment, with CCG intervention rates ranging from 4 to 195 interventions per 100 000 per year. There was a slight reduction in variation after EBI (2–173 per 100 000 per year), but this came at the cost of the median rate falling further behind the estimated ideal, and the magnitude of variation remains remarkable. This equates to a range in the number of procedures performed annually of between 64 and 1384 for the average-sized vascular surgical service serving a population of around 800 000.

Some CCGs saw higher than expected procedure rates. The incidence of this was greatest in (but not limited to) green CCGs. There are several potential explanations for this. The first is that some may be related to overtreatment (treatment of patients outside of the NICE guidelines). A second explanation is related to limitations in coding. The advent of minimally invasive treatment has resulted in pathway changes. For example, previously a patient requiring treatment to both legs would have had this done as a single procedure under general anaesthetic. A two-stage procedure for bilateral disease is now more common with local anaesthetic techniques. It is possible that this may be differentially coded as single or multiple consultant spells for the purposes of HES data. In addition, despite best evidence to the contrary^12,13^, varicose tributaries are treated variably during the first procedure and, in some centres, patients return for secondary treatments, again potentially inflating the intervention rate. The final possibility is that the predicted intervention rates may not be correct, the actual figure being higher. Some of the assumptions made in the NICE model may have tended to favour a more conservative estimate of intervention rate. Further research is required to understand this finding and its underlying causes.

There is extensive geographical variation in numbers of varicose vein procedures performed^14^. This study has demonstrated the significant association between level of CCG policy compliance and intervention rate but, interestingly, it accounted for only 17 per cent of the variation observed. There was also significant crossover, with some red CCGs having higher intervention rates than some green ones. This suggests that there are other significant factors involved. One factor may be related to CCG processes that make the practicality of commissioning more or less restrictive than is apparent from the published policy. For example, some CCGs may use a case review system via individual funding request applications, which may not be applied in line with the published policy, making it more restrictive. Alternatively, CCGs may use a form-based application where, as long as the correct boxes are ticked, funding is granted, making it potentially less restrictive. Over 50 per cent of CCGs limit based on clinical severity; the reason for this is unclear, but perceived affordability of varicose vein interventions could be a potential reason. Other factors may occur throughout the patient pathway that are related to: interpretation of the EBI document, education and awareness of the local patient population, alteration in the rate at which patients present to healthcare services, the approach and education of primary care, a commonly held belief that varicose veins are simply of cosmetic concern, or that venous leg ulcers are referred inappropriately to other services. Similarly, there may be differing attitudes from vascular services towards this patient group. More likely, however, is that the weight of the critical lifesaving and limb salvage arterial workload on the background of a workforce crisis is a contributing factor^15^. There are few data available to unpick these issues at present, but this area is worthy of further research.

The study limitations include the assumptions made in both the NICE commissioning model and the models used in this analysis. These assumptions, however, have tended towards minimizing the size of the challenges and healthcare under provision. Limitations in HES data coding may have influenced the findings.

NHS England and the Secretary of State for Health and Social Care share a legal duty to promote a comprehensive health service in England, in accordance with the National Health Service Act 2006 (as amended by the Health and Social Care Act 2021)^16^. This is supported by the commitments set out in the NHS Long Term Plan (2019–2020), NHS Long Term Plan Implementation Framework, and criteria set by the Secretary of State to address and reduce health inequalities^17,18^. The NHS Long Term Plan lays out specific ambitions for the NHS to take a ‘more concerted and systematic approach to reducing health inequalities’ by addressing unwarranted variation in access to, experience of, and outcomes from, treatment and care^17^. It is outlined that Sustainability and Transformation Partnerships (STPs) and Integrated Care Systems (ICS) will have the responsibility to strategize and deliver the NHS Long Term Plan objectives and respond to local needs to reduce local health inequalities and unwarranted variation in access to services and care. In geographical areas of poor policy compliance and low intervention rates, STP/ICS should concentrate their efforts on the services, workforce, and finances needed to deliver venous care in line with NICE CG168. It is not clear how STP/ICS are proposed to succeed where CCGs have failed. Furthermore, the recovery of services to prepandemic levels stands to be an enormous challenge nationally, which will be furthered by the need to improve on these levels. The stakes are high; patients have been denied the right to access NICE-recommended treatment under the NHS constitution for almost a decade^19^, there has been a dramatic curtailment in venous services during the SARS-CoV-2 pandemic, and the numbers of patients in need of treatment, the lost QALYs, and the lost benefit of healthcare expenditure is spiralling.

## Supplementary Material

znac392_Supplementary_Data

## Data Availability

Additional data are available on request to the corresponding author.
