# Supplementary material for: Provision of NICE-recommended varicose vein treatment in the NHS
Source: Br J Surg. 2022 Nov 30;110(2):225–32. doi: 10.1093/bjs/znac392 (PMC10364503; doi:10.1093/bjs/znac392)
Supplement: znac392_Supplementary_Data [file znac392_supplementary_data.docx]

**Title**

**The provision of NICE recommended Varicose Veins Treatment in the NHS**

Authors

Louise Helen Hitchman^1^, Abduraheem Mohamed ^1,2^, George Edward Smith ^1,2^, Sean Pymer ^1,2^, Ian Clifford Chetter^1,2^, James Forsyth,^3^ Dan Carradice^1,2^

^1^ Academic Vascular Surgery Unit, Hull York Medical School

^2^Academic Vascular Surgery Unit, Hull University Teaching Hospitals NHS Trust

^3^Department of Vascular Surgery, Leeds University Teaching Hospital NHS Trust

**Corresponding author.**

Ms. Louise Hitchman (ORCiD: 0000-0001-9681-6749), Academic Vascular Surgery Unit, 2^nd^ Floor Allam Building, Hull Royal Infirmary, Anlaby Road, Hull, HU3 2JZ, l.hitchman@nhs.net

**Supplementary Materials - Index**

| **Supplementary Methods** |  |
| --- | --- |
| NICE Costing Template | *pag. 2* |
| Hospital Episode Statistics Codes | *pag. 3* |
| **Supplementary Results** |  |
| Net Health Benefit Estimation Tables | *pag. 5* |
|  |  |
|  |  |
|  |  |
|  |  |
|  |  |
|  |  |
|  |  |
|  |  |
|  |  |

**Supplementary Methods**

*NICE Costing Template*

The NICE Costing Template for NICE CG168 Guideline: Varicose veins in the leg: diagnosis and management of varicose veins.

*Hospital Episode Statistics Codes*

The following codes were used to extract data on the number of varicose vein episodes per CCG in 2017/2018, 2018/2019 and 2019/2020.

**OPCS code**

- L841: Combined operations on primary long saphenous vein

- L842: Combined operations on primary short saphenous vein

- L843: Combined operations on primary long and short saphenous vein

- L844: Combined operations on recurrent long saphenous vein

- L845: Combined operations on recurrent short saphenous vein

- L846: Combined operations on recurrent long and short saphenous vein

- L848: Other specified combined operations on varicose vein of leg

- L849: Unspecified combined operations on varicose vein of leg

- L851: Ligation of long saphenous vein

- L852: Ligation of short saphenous vein

- L853: Ligation of recurrent varicose vein of leg

- L858: Other specified ligation of varicose vein of leg

- L859: Unspecified ligation of varicose vein of leg

- L861: Injection of sclerosing substance into varicose vein of leg NEC

- L862: Ultrasound guided foam sclerotherapy for varicose vein of leg

- L863: Injection of glue into varicose vein of leg

- L868: Other specified injection into varicose vein of leg

- L869: Unspecified injection into varicose vein of leg

- L871: Stripping of long saphenous vein

- L872: Stripping of short saphenous vein

- L873: Stripping of varicose vein of leg NEC

- L874: Avulsion of varicose vein of leg

- L875: Local excision of varicose vein of leg

- L876: Incision of varicose vein of leg

- L877: Transilluminated powered phlebectomy of varicose vein of leg

- L878: Other specified other operations on varicose vein of leg

- L879: Unspecified other operations on varicose vein of leg

- L881: Percutaneous transluminal laser ablation of long saphenous vein

- L882: Radiofrequency ablation of varicose vein of leg

- L883: Percutaneous transluminal laser ablation of varicose vein of leg NEC

- L888: Other specified transluminal operations on varicose vein of leg

- L889: Unspecified transluminal operations on varicose vein of leg

**Supplementary Results**

*Net Health Benefit Estimation Tables*

- Not Adjusted

|  | Estimated Deficit in Number of Procedures |  |
| --- | --- | --- |
| 2017/2018 | 38790 |  |
| 2018/2019 | 40029 |  |
| 2019/2020 | 43040 |  |
| Estimated Total CCG Saving | £95,050,105.80* |  |
|  | Estimated Loss in Health Benefit (QALYs) by year | Estimated Cumulative Loss in Health Benefit (QALYs) over the period |
| 2017/2018 | 2094.66 | 2094.66 |
| 2018/2019 | 2161.57 | 6350.89 |
| 2019/2020 | 2324.17 | 12931.28 |
| Estimated Loss in Health Benefit | £258,625,558.80^†^ | |
| Estimated Loss in Net Health Benefit | £163,575,453.00 | |

QALY=Quality Adjusted Life Year

*Assuming the cost of a consultant to provide a endovenous ablation procedure of £780, based on the NICE costing template

^†^Where the willingness to pay threshold is £20,000 per 1 QALY

- Adjusted for >110%

|  | Estimated Deficit in Number of Procedures |  | Number of procedures over 110% of the expected intervention rate |
| --- | --- | --- | --- |
| 2017/2018 | 40464 | 2017/2018 | 1674 |
| 2018/2019 | 41867 | 2018/2019 | 1838 |
| 2019/2020 | 44058 | 2019/2020 | 1018 |
| Estimated Total CCG Saving | £98,583,505.80* | Total. no procedures outside CG168 | 4530 |
|  | Estimated Loss in Health Benefit (QALYs) by year |  | Estimated Cumulative Loss in Health Benefit (QALYs) over the period |
| 2017/2018 | 2185.056 |  | 2185.056 |
| 2018/2019 | 2260.818 |  | 6630.93 |
| 2019/2020 | 2379.14 |  | 13455.94 |
| Estimated Loss in Health Benefit | £269,118,838.80 | | |
| Procedures cost waste | £3,533,400.00 | | |
| Estimated Loss in Net Health Benefit | £174,068,733.00 | | |

*Assuming the cost of a consultant to provide a endovenous ablation procedure of £780, based on the NICE costing template

^†^Where the willingness to pay threshold is £20,000 per 1 QALY
